# Medication misuse and illicit substance use among palliative care patients in German palliative care units– an evaluation from the perspective of palliative care providers

**DOI:** 10.1186/s13722-025-00560-3

**Published:** 2025-03-31

**Authors:** Jannis Eersink, Julian Maul, Nils Heuser, Astrid Morin, Martin Gschnell, Christian Volberg

**Affiliations:** 1 Department of Anaesthesiology & Intensive Care Medicine, Philipps University of Marburg, Marburg, Germany; 2 Department of Dermatology and Allergology, Faculty of Medicine, Philipps University of Marburg, Marburg, Germany; 3 Research Group Medical Ethics, Faculty of Medicine, Philipps University of Marburg, Marburg, Germany; 4 Department of Anaesthesiology and Intensive Care Medicine, Research Group Medical Ethics, Philipps University of Marburg, Baldingerstraße, 35043 Marburg, Germany

**Keywords:** Substance misuse, Palliative care, Addiction, Potential risk, Substance use disorder

## Abstract

**Background:**

Palliative care focuses on controlling symptoms and improving the patient’s quality of life. To achieve this, medications with addictive potential are often used. There have been various case reports of substance misuse in palliative care. This study aims to explore how practitioners perceive the issue and management of substance misuse in palliative care patients.

**Materials and methods:**

Following an extensive literature review, a 23-question questionnaire was developed to assess attitudes and practices related to substance misuse in palliative care and distributed to all German palliative care units (PCUs) listed on the website of the German Society for Palliative Medicine (*n* = 334).

**Results:**

A total of 116 responses from PCUs (34.7%) were included in the analysis. Of these, 49.1% estimated that approximately 1–5% of their patients suffer from medication-related substance misuse. Most respondents (72.4%) assumed that 1–5% of their patients use illicit substances. In addition, 62.9% of the PCUs do not screen their patients for substance use disorders, while only 0.9% report doing so regularly. In the case of addiction problems, 55.2% of the PCUs do not implement any specific measures. Most respondents described their approach to prescribing medications with potential for substance misuse as liberal (71.6%) or very liberal (12.9%). Furthermore, 78.4% reported that the addictive potential of a medication has little or no influence on their prescribing decisions. Finally, 67.2% of participants expressed a desire for more education about addiction in palliative care.

**Discussion:**

The data collected in our study indicate that, from the perspective of palliative care professionals, substance use disorders are not perceived as a significant problem for patients receiving inpatient palliative care. However, we found that most PCUs do not screen their patients for substance misuse, suggesting that most practitioners may not have a comprehensive view of the actual number of dependent patients. Further research is therefore needed to obtain reliable data on the number of patients with substance use disorders in palliative care and to determine the point at which substance misuse is caused by medical prescription.

## Introduction

In Germany, the provision of palliative care for patients with incurable diseases is based on a two-level care system. Primary care is provided by general practitioners, nursing services and hospital physicians at a general palliative care level. The second level includes palliative care units (PCUs) attached to hospitals where acute medical problems can be treated. In addition, there are specialized outpatient palliative care services (German abbreviation: SAPV), which provide palliative care at home, and hospices, where people with a low life expectancy can live and receive extensive palliative care until they die [1]. The provision of specialized palliative care is a relatively new medical specialization in Germany, beginning with the opening of the first PCU at the University Hospital of Cologne in 1983 [2]. Since then, it has continuously evolved and plays a central role in the care of severely ill and dying patients. Symptom control is one of the overarching goals of palliative medical support [3]. In particular, the relief of pain, dyspnea, and anxiety are prioritized [4].

Commonly used medication include metamizole as a non-opioid analgesic, hydromorphone, morphine and fentanyl as opioid analgesics, and pregabalin as a co-analgesic [5, 6]. Other medications that may contribute to substance misuse are also used. These include Benzodiazepines, various opioids, and psychopharmaceuticals [4–7]. In palliative medicine, these medications are often liberally administered, as patients with a high symptom burden typically have a short remaining time to live. They should spend this time with the best possible quality of life and without stressful symptoms.

However, new therapies (e.g., immunotherapy or checkpoint inhibition) have significantly extended the survival periods of patients with incurable cancer, which means that potent medicines for symptom control are also taken for a much longer period [8–12]. A particularly striking example is the treatment of malignant melanoma, where 43% of patients are still alive at 10-year follow-up thanks to the combination therapy of nivolumab with ipilimumab [13]. Although palliative care is often associated with oncology patients, more and more patients with chronic medical conditions (e.g. COPD, heart failure) and neurological conditions (e.g. Parkinson’s disease, multiple sclerosis) are receiving palliative care. It is often difficult to predict how long palliative care will last, especially with improved oncological therapies, but also in the case of the chronically ill [14–16]. This may lead to a risk of iatrogenic substance misuse, especially in susceptible individuals (e.g. those with a history of addiction problems). Several patients with medication misuse have been recently cared on our PCU, where the quality of life for the affected individuals and their families was severely reduced. It remains unclear whether this was a coincidence or if the problem is inherent in palliative care. There is little international literature on this topic [17–19]. Individual studies show that general screening for addiction problems in palliative care patients is quite rare [20]. Therefore, the exact number of patients affected can usually only be estimated using data from studies. A 2016 literature review found that one in five cancer patients was at risk of opioid misuse, while another study found that the figure was 30.8% [21, 22]. This needs to be distinguished from patients with known substance use disorders, who often have a large number of co-morbidities and require a completely different type of care [23].

As there is a paucity of data from Germany on this topic, this publication aims to explore the extent to which addiction is perceived as a problem by palliative care professionals in Germany, whether targeted screening for substance misuse occurs, and whether measures are taken against existing addictions.

## Method

After an extensive literature review on the topic, the study team developed a questionnaire with a total of 23 questions. This questionnaire was checked for comprehensibility by five uninvolved colleagues before the start of the study. After the positive vote of the local ethics board from October 7th, 2022 (file number 139/22; ethics board of the Philipps University of Marburg, Germany), and the registration in the German register of clinical trials (DRKS-ID: DRKS00030427, registration date 24.10.2022), the questionnaire was sent on November 17th, 2022, to all German palliative care units for children and adults listed on the website of the German Society for Palliative Medicine (*n* = 334) [24, 25]. These are the inpatient PCUs mentioned above, which are attached to a hospital as described in the introduction. As a primary outcome, the questionnaire aimed to assess how caregivers in PCUs perceive the issue of medication misuse and illicit drug dependence among their patients. In addition, secondary outcomes included data on PCU demographics and team attitudes and prescribing practices regarding medications with potential for substance misuse. The survey was conducted anonymously, with a prepaid return envelope enclosed for the responses.

All questionnaires received by March 31st, 2023, were included in the analysis. The evaluation was primarily descriptive, and significance testing for group differences was performed using the Chi-square test with a significance level of *p* < 0.05. Open-ended text responses were included in the analysis, where legible. Medications were standardized to uniform active ingredient names. Microsoft^®^ Excel Version 16.68 was used for data analysis and processing.

## Results

In total, 116 out of the 334 (34.7%) contacted PCUs responded. Of these, 87.1% of the questionnaires were filled out by the leading physician of the PCU. Most PCUs were located in medium-sized cities (population < 100.000 inhabitants) (40.5%) or large cities (pop. > 100.000 inhabitants) (35.3%). Most PCUs cared for up to 300 patients per year, and 97.4% exclusively treated adult patients (see Table 1).

**Table 1:** Survey data on the care of patients with problematic medication and drug use

	*n*	%
**Who fills out the questionnaire?**		
Responsible/leading physician	101	87.1
Nursing management	11	9.5
Healthcare and nursing assistants	1	0.9
Ward physician	2	1.7
Nurse	1	0.9
**Which residential area applies to your supply area?**		
Metropole (pop. > 1 Mio.)	9	7.8
Large city (pop. > 100.000)	41	35.3
Medium-sized city (pop. < 100.000)	47	40.5
Small town, rural area (pop. < 20.000)	20	17.2
**How many patients do you care for on average per year?**		
1-100	3	2.6
101–200	29	25.0
201–300	44	37.9
301–400	23	19.8
> 400	16	13.8
Not specified	1	0.9
**Do you care for children or adults?**		
Adults	113	97.4
Children	1	0.9
Both	2	1.7
**Do you have doctors on your team who have an additional qualification in “Addiction Medicine”?**		
Yes	11	9.5
No	104	89.7
Not specified	1	0.9
**If yes**, **number of doctors in the team with additional qualification**:		
1	7	6.0
2	1	0.9
3	1	0.9
4	1	0.9
5	0	0.0
6	1	0.9
**How high do you estimate the proportion of your patients with medication misuse?**		
0%	3	2.6
1–5%	57	49.1
6–10%	34	29.3
11–20%	9	7.8
21–30%	6	5.2
> 30%	6	5.2
Not specified	1	0.9
**What percentage of patients with medication misuse do you consider to be iatrogenically induced?**		
0%	5	4.3
< 10%	43	37.1
10–25%	25	21.6
26–50%	20	17.2
51–75%	14	12.1
> 75%	8	6.9
Not specified	1	0.9
**How high do you estimate the proportion of your patients who use illegal drugs?**		
0%	31	26.7
1–5%	84	72.4
6–10%	1	0.9
11–20%	0	0.0
21–30%	0	0.0
> 30%	0	0.0
**Do you have the impression that the number of palliative patients with medication misuse and/or illicit drug abuse has increased in the last 10 years?**		
Yes	15	12.9
No	74	63.8
I am not sure	26	22.4
Not specified	1	0.9

In total, 9.5% of the participating PCUs had at least one doctor with an additional qualification in “Addiction Medicine” on their team.

Almost half (49.1%) of respondents estimated that 1–5% of their patients misuse potentially addictive medicines. An additional 29.3% estimated this figure to be between 6 and 10% of their patients. According to 37.1% of respondents, this medication misuse is iatrogenically induced in less than 10% of patients. However, 21.6% indicated that this applies to 10–25% of their patients (see Table 1).

**Table 2:** Attitudes and practices regarding the prescription of addictive medications in palliative care

	*n*	%
**How would you describe the attitude in your palliative care unit team towards prescribing medications with addictive potential?**		
Very liberal	15	12.9
Liberal	83	71.6
Restrictive	16	13.8
Very restrictive	1	0.9
Not specified	1	0.9
**To what extent does the potential risk of addiction influence your decision to prescribe a medication?**		
Very strong	3	2.6
Strong	19	16.4
Little	71	61.2
Very little	20	17.2
Not specified	3	2.6
**Is it documented exactly how many and which medications the patient is taking?**		
Yes	116	100
No	0	0
**Which routes of administration do you use for fast-acting opioids?**		
None	0	0
Nasal spray	60	51.7
Buccal	96	82.8
Subcutaneous	106	91.4
Intravenous	102	87.9
Rectal	0	0.0
Inhalative	6	5.2
**Would you like to see more education/training about addiction in palliative care?**		
Yes	78	67.2
No	36	31.0
Not specified	2	1.7

**Fig. 1:**
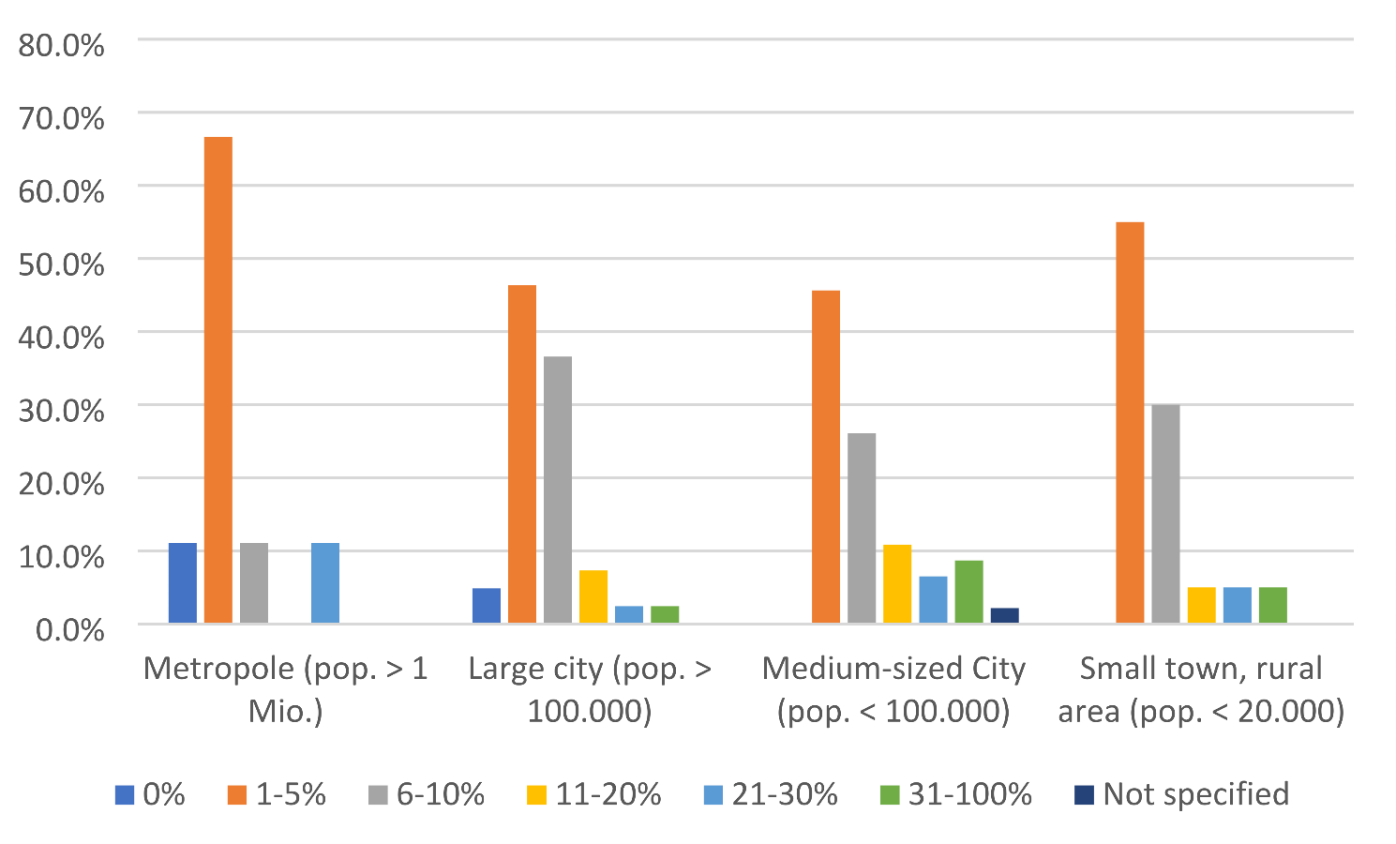
Estimated medication misuse by living situation

The estimated number of patients with medication misuse by place of residence is shown in Fig. 1. It appears that PCUs in metropolitan areas are less likely to report that patients are misusing medicines.

As a substance class with particularly high potential for medication misuse, 63.8% of PCUs mentioned benzodiazepines, and 40.5% cited opioids.

The majority of respondents (72.4%) stated that between 1 and 5% of their patients suffer from drug addiction. In Germany, drugs are defined as substances that produce some kind of high and are addictive, regardless of whether they are legal (e.g. alcohol or cannabis) or illegal (e.g. cocaine or heroin). This definition does not usually include medicines. Only one PCU reported that more than 5% of their patients are affected (see Table 1). 63.8% believed that the proportion of palliative care patients with drug or medication dependence had not increased over the last ten years.

**Fig. 2:**
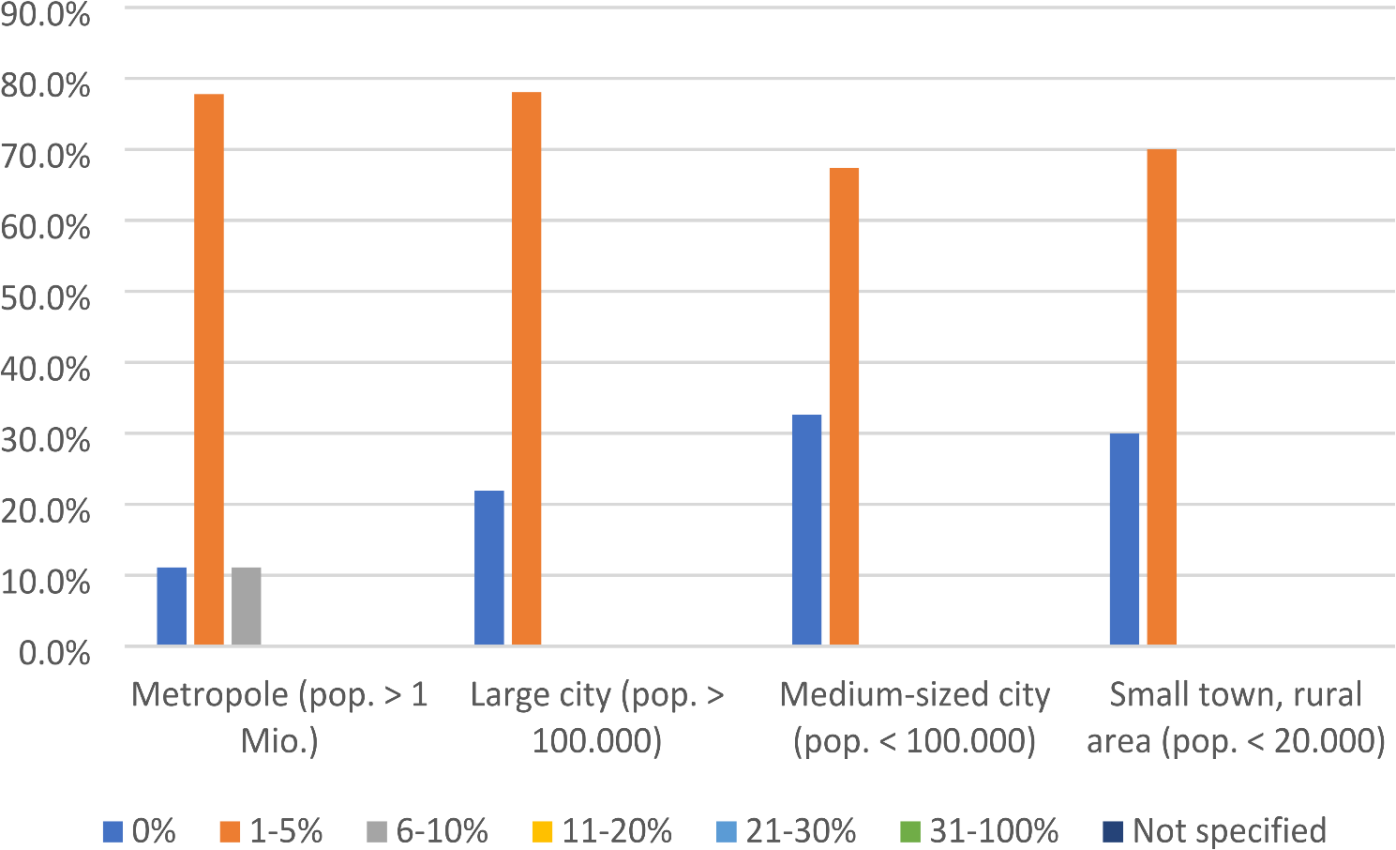
Estimated drug dependence by living situation

In contrast to the responses for medication misuse, PCUs in metropolitan areas and large cities were significantly more likely to report that their patients suffered from drug dependence (*p* = 0.03) (see Fig. 2).

In this context it is important to note that 62.9% of PCUs do not screen their patients for critical medication or drug use. Only one unit regularly screens its patients, and 23.3% screen in cases where substance misuse is suspected (see Table 3). Among the PCUs that do screen, the Short Questionnaire for Drug Use (comparable to the DRUG USE QUESTIONNAIRE (DAST)) or other screening methods are most used (both 30.6%). No PCU routinely screens relatives for substance misuse, with 89.7% never doing so.

**Table 3:** Screening and treatment practices for patients with critical medication or drug use

	*n*	%
**Do you routinely screen your patients for the presence of critical medication and/or drug use?**		
No	73	62.9
Only on admission/start of care	13	11.2
Only if substance misuse is suspected	27	23.3
Regularly	1	0.9
Not specified	2	1.7
**If yes**, **which screening questionnaire(s) do you use?**		
ASSIST (Alcohol, smoking and substance involvement screening test)	8	6.9
DIPS (Diagnostic interview for mental disorders)	4	3.4
KFM (Short questionnaire for medication use)	11	9.5
LBC (Lippstadt benzo check)	2	1.7
SKID-II (Structured Clinical Interview for DSM-IV)	0	0.0
Other	11	9.5
**Do you screen your patients’ relatives for the presence of substance misuse?**		
Yes, regularly	0	0.0
Yes, but only if substance misuse is suspected	12	10.3
No	104	89.7
**Do you carry out anti-addiction measures for patients with addiction problems?**		
Yes	51	44.0
No	64	55.2
Not specified	1	0.9

55.2% of the PCUs reported that they do not initiate therapy for treatment of substance use disorders when it is detected. If action is taken, many respondents indicated in the open-ended response section that this typically involves a referral or consultation with a psychiatrist. Some PCUs manage detoxification independently, often through educational discussions and dose reductions.

A large portion of respondents described their team’s attitude towards prescribing medications with substance misuse potential as liberal (71.6%) to very liberal (12.9%) (see Table 2). Accordingly, the majority reported that the potential danger of substance misuse influences their decision to prescribe a medication little (61.2%) to very little (17.2%) (see Table 2).

All PCUs reported that they document the quantity and types of medications a patient is taking. For the administration of fast-acting opioids, the preferred methods are subcutaneous (91.4%), intravenous (87.9%), and buccal (82.8%).

Most respondents (67.2%) stated that they are interested in more educational opportunities on the topic of addiction in palliative medicine.

In response to the final question about whether the study participants had anything else to share, some noted that addiction issues in palliative medicine should be differentiated more precisely according to the patient’s current phase of care. They believe that the patient´s remaining life expectancy significantly impacts in how substance use disorders should be approached. Other respondents suggested a more thorough investigation into whether patients in palliative care develop medication misuse during palliative care treatment or whether this occurs during the curative phase. Additional feedback expressed concerns about focusing too much on this issue, as many patients already fear becoming dependent on opioids. Participants were concerned that further research could intensify these fears.

## Discussion

The data we collected align with findings from previous studies. For instance, a study conducted in 2007 reported that up to 7.7% of all cancer patients suffer from medication addiction, a figure that corresponds with the estimates provided by the palliative care units in our survey [26]. However, a critical consideration when comparing these data is that the authors of the earlier study did not differentiate between patients in curative and palliative treatment.

Patients admitted to inpatient palliative care are typically those for whom general outpatient palliative measures are insufficient due to complex symptom burdens [27]. These patients often receive high doses of potent analgesics and other medications with potential for substance misuse. Consequently, the number of medications prescribed to palliative patients tends to increase due to the greater need for symptom control, while the use of non-palliative medications (e.g. statins) decreases towards the end of life [28].

Palliative care patients frequently consume high doses of analgesics because alternative methods of pain reduction, such as physiotherapy, cannot be applied adequately, or because they use these medications to cope with their terminal illness or loneliness [29, 30].

It is important to note that while current reports are mainly anecdotal [17–19], there is a significant number of patients being treated with the aforementioned medications. Currently, 10–12% of all terminally ill patients in Germany require specialized palliative care [3]. These numbers are expected to rise in the future. The German S3 guideline on palliative medicine suggests that the previously estimated figures of 10–15% are likely underestimated and that in the future, 25–65% of terminally ill patients will need specialized palliative care [27]. These projections are partly based on a 2014 study which predicts that by increasingly including patients without cancer diagnoses in palliative care, the percentage of terminally ill requiring such care could rise to even higher rates [31].

An US American study from 2012 found a significantly higher number of patients with critical medication misuse than we determined. In this study, 77.2% of all surveyed palliative care physicians reported seeing a patient with critical medication or drug use in the past two weeks. 43.9% reported seeing patients with critical opioid use [32]. A study conducted three years later supported these numbers, showing that in a cross-sectional study, 46% of patients in an oncological clinic had critical screening values [33]. However, it is questionable to what extent these data from the US can be transferred to German palliative care patients, especially in light of the opioid crisis in America, which has been partly supported by doctors [34, 35]. Although Germany does not have an opioid crisis like the United States of America, the use of opioids and other potentially addictive medications should be viewed critically [36]. The INTERREG study revealed that 30% of hospital inpatients consume excessive alcohol, and benzodiazepines were detected in a third of all nursing home residents although 68% of them had not been prescribed a benzodiazepine [37].

Further studies are necessary to determine whether the figures in Germany are indeed lower, or whether caregivers pay less attention to their patients’ substance misuse. In our study, 62.9% of participants reported that they never test their patients for critical drug or medication use, even if there is an indication for it. Only one PCU indicated that they regularly screen their patients. In contrast, a US study of outpatient palliative care physicians found that 71% used urine tests to screen their patients for substance misuse [38]. It is therefore likely that caregivers in Germany are currently unaware of how many of their patients have critical drug or medication misuse.

Understanding the prevalence of these issues is crucial for providing optimal palliative care. Palliative care should prevent overdoses and dependencies, as these can affect the quality of life. On the other hand, it is also known that many patients are not sufficiently treated for pain [39, 40]. The desire for more research on this topic was repeatedly mentioned in the free-text responses of our survey. In particular, studies investigating whether patients with substance misuse in palliative care developed this dependency through palliative care or had already received it from other caregivers, possibly in a curative setting, are of interest to some of the study participants.

On the other hand, nearly half of all patients with terminal cancer are not adequately treated with analgesics [39]. One reason for this is that many patients are afraid of becoming dependent on pain medication, especially opioids [41]. Even those patients who are treated with potent analgesics often do not receive them in sufficient doses. Although 97% of all palliative care patients are regularly prescribed opioids in the last weeks of life, 25% of these patients reported that their pain was not sufficiently reduced by this treatment [40]. As the prescription of opioids and other potentially addictive medicines is well regulated in Germany, care must be taken to ensure that patients are not under-treated for fear of addiction. Some palliative care physicians of our survey considered this fear of undersupply to be more dangerous than the possibility of substance misuse, which is in line with findings of another survey [42].

It is therefore urgently necessary to have reliable data on substance misuse on the one hand and insufficient treatment of palliative care patients on the other hand. Above all, treating physicians need appropriate screening tools to reliably identify patients with a high risk of developing a substance misuse. This could be achieved through the regular use of screening questionnaires for addiction disorders. As there is currently a lack of data to make firm recommendations, the general advice from our findings could be that the use of potentially addictive medications should be critically reviewed, and patients should be continuously monitored for drug or medication dependence. Particular attention should be paid to patients who respond well to their cancer treatment and enter a “chronic palliative phase”. Pain management for these patients should ideally be based on guidelines on the long-term use of opioids in patients with chronic pain (in Germany: LONTS) [43]. However, this should not lead to palliative care patients being undertreated out of fear of substance misuse. Most palliative care specialists do not see themselves as experts in substance use disorders, although many colleagues consider them experts in these issues [44]. Palliative care physicians should therefore develop expertise in addiction medicine. This way, they can optimize and, if necessary, intensify the therapy of their patients, and more effectively recognize and protect potentially at-risk patients.

### Strengths and limitations

This survey is the first to investigate the extent to which substance use disorders are perceived as a problem in German palliative care units and the measures taken to prevent it. A main finding is that two-thirds of German palliative care units do not screen their patients for addiction and that the majority of participants would like more information on this topic in the form of further training or scientific education. On the other hand, this study provides only a preliminary overview of the relationship between palliative care and medication misuse and drug dependence. Particularly since the data are based on estimations by caregivers, the study´s validity is limited. Further studies are needed to collect data directly from patients to improve accuracy and to compare how the participants’ perceptions match reality. Moreover, this study cannot determine how medication misuse affects the life satisfaction of the affected patients. Additionally, the data do not clarify whether medication misuse was acquired through palliative care or was already present beforehand.

## Conclusion

As the number of ‘chronic’ palliative care patients continues to increase due to modern therapeutic options in cancer therapy and therefor longer survival times are achieved, palliative care physicians should pay closer attention to issues of substance misuse among their patients. Extended therapy durations can increase the risk of substance misuse for susceptible individuals. Since individuals often do not know, if a patient has a dependency problem, conducting more comprehensive screenings would provide a better insight into the benefit-risk ratio of their own treatment. Furthermore, training and further education on this subject are essential for raising awareness among palliative care physicians. This approach would enable them to better identify risks while maximizing the potential for appropriate symptom control for their patients.

## Data Availability

The datasets used are available from the corresponding author on reasonable request.
